# Psychoactive substances as a last resort—a qualitative study of self-treatment of migraine and cluster headaches

**DOI:** 10.1186/s12954-017-0186-6

**Published:** 2017-09-05

**Authors:** Martin Andersson, Mari Persson, Anette Kjellgren

**Affiliations:** Department of Psychology, Karlstad University, SE-651 88 Karlstad, Sweden

**Keywords:** Migraine, Cluster headache, Alternative treatments, Psychedelic tryptamines, Harm reduction, Internet support groups, Discussion forum

## Abstract

**Background:**

Treatment resistant cluster headache and migraine patients are exploring alternative treatments online. The aim of this study was to improve comprehension regarding the use of non-established or alternative pharmacological treatments used by sufferers of cluster headaches and migraines.

**Methods:**

A qualitative thematic analysis of the users’ own accounts presented in online forum discussions were conducted. The forum boards https://shroomery.org/, http://bluelight.org, and https://clusterbusters.org/ met the inclusion criteria and were used for the study.

**Results:**

The analysis resulted in six themes: *a desperate need for effective treatments*; *the role of the forum—finding alternative treatments and community support*; *alternative treatment substances*; *dosage and regimens*; *effects and treatment results*; and *adverse effects*. The results provide an insight into why, how, and by which substances and methods sufferers seek relief from cluster headache and migraines.

**Conclusions:**

These patients are in a desperate and vulnerable situation, and illicit psychoactive substances are often considered a last resort. There appeared to be little or no interest in psychoactive effects per se as these were rather tolerated or avoided by using sub-psychoactive doses. Primarily, psilocybin, lysergic acid diethylamide, and related psychedelic tryptamines were reportedly effective for both prophylactic and acute treatment of cluster headache and migraines. Treatment results with cannabis were more unpredictable. No severe adverse events were reported, but it was observed how desperation sometimes spurred risky behavior when obtaining and testing various treatment alternatives. The forum discourse mainly revolved around maximizing treatment results and minimizing potential harms.

## Background

Migraine and cluster headache (CH) are prevailing, episodic, often chronic headache disorders that have a considerable impact on the individual and society [1]. Especially, migraine with a prevalence of nearly 15% worldwide is a significant cause of disability and notably burdens medical costs and loss of productivity. Cluster headaches are a rarer but particularly painful and debilitating form of headache disorder with a prevalence around 1 in 1000 individuals [2]. While there are numerous treatment practices for headache disorders, none are ideal and most exhibits unsatisfactory effectiveness, tolerability, or patient adherence [1]. There are presently no pharmacological treatments available specifically developed for CH. The currently used methods originated as treatments for other indications and were found helpful in CH by chance [3]. CH is known to be sometimes resistant to the conventional therapies (around 20% in chronic cases of CH) [4, 5]. Considering that CH is one of the most intense and disabling pain conditions known, the urgency of the circumstances has led care providers and patients to try unusual or experimental remedies [6]. However, CH patients sometimes fail to respond also to the more experimental methods used in clinical practice [6].

Dissatisfaction with conventional therapies and adverse effects can often motivate the use of complementary and alternative medicine (CAM) [7]. Also, the general interest in CAM has seen an upsurge over the past decades in both the USA and Europe [7, 8]. There is currently a growing interest and some evidence supporting various complementary or alternative medicine treatments of headache disorders [7, 9]. One controversial, but increasingly reported effective treatment is the use of illicit psychoactive (psychedelic) tryptamines like lysergic acid diethylamide (LSD) and psilocybin. A few studies, as well as extensive anecdotal support, have indicated the effectiveness of psychedelic tryptamines for the treatment of both CH and migraines [3, 6, 7, 9–12]. These substances are structurally similar (indole alkaloids) to the triptans currently prescribed for the treatment of CH. Even so, the prescribed non-psychoactive triptans do not abort cluster episodes or prolong remission periods as psilocybin or LSD reportedly does [13]. Schindler et al. state that the combination of high efficiency and low rate of adverse effects observed with the psychedelic tryptamines is not seen in any of the currently used treatments [9]. However, some reports on the non-psychoactive LSD analog BOL-148 have shown equally promising results for the treatment of cluster headache with similarly reported low rates of adverse effects [9, 13–15]. BOL-148 is currently not available for use in clinical practice.

A few published studies [10, 16] and rich anecdotal supports also indicate the effectiveness of cannabis for alleviating headaches, but to our knowledge, no proper clinical trials are currently available. Historically, cannabis was well-regarded as an acute, as well as prophylactic, treatment for headache disorders and was included in the major pharmacopeias of the second half of the nineteenth century [10]. The illegal status of cannabinoids and psychedelics has critically hindered medical research, and there are currently no blinded studies on headache patients so true effectiveness can be determined [10]. To improve understanding of the effects and possible benefits or harms of scarcely researched substances, Internet discussion forums, and the users’ own accounts of their experiences, have proven to be a valuable source for surprisingly accurate early research data when clinical trials are not available [17–22].

Increasingly, the Internet serves as a primary source for information on personal health concerns. In the current digital landscape, patients and caregivers now have easy access to each other and Internet support groups (ISG) are formed around most medical conditions. Almost a quarter (23%) of those with long-term conditions reportedly uses the Internet to seek out peers [4]. As the web transformed from the more static and hierarchical structures of the early days to the emergence of a co-creational social media environment (Web 2.0 technologies), there is an ongoing shift from merely searching for health information to that of reciprocally producing and evaluating content. A corresponding municipally based knowledge production (“citizen science”) is observed in the recreational drug communities online [21]. There is also a considerable overlap between the psychoactive drug discussions and the health communities online as psychoactive drugs sometimes are utilized as attempted self-medication [19, 20, 23]. This overlap is present to a high degree amongst the headache disorder patient groups exploring alternative treatments online as these discussions commonly focus on medicating with various psychoactive substances. In line with our previous drug discussion studies, we applied a similar approach using thematic analysis of forum discussions by sufferers of headache disorders as a basis for the present study.

### Aim

The aim of this study was to improve comprehension regarding the use of non-established or alternative pharmacological treatments used by sufferers of cluster headaches and migraines.

## Methods

### Data collection

To find suitable forums, Google searches using the keywords “cluster headache discussion forums” and “migraine discussion forums” were conducted on April 18, 2016, and supplemented by a search with keywords “drug discussion Forum” on April 19, 2016. Searches resulted in 13,600, 53,600, and 574,000 hits, respectively, and for each search, the 100 first hits were further examined. A total of 10 websites contained discussion forums and gave hits on searches with keywords “cluster,” “headache,” or “migraine.” Amongst these, two sites were focused on alternative treatments rather than established medical treatments and were chosen for the study: *Shroomery.org* and *Bluelight.org*. A third forum, *Clusterbusters.org*, was found through references from the first two forums. The internal search function of the forums was then used to perform the searches utilized for the collection of data. Table 1 presents the number of topics with keyword “treatment cluster”; Table 2 entails topics from keyword “treatment migraine.” To limit the data to a manageable size, the selection was restricted to topics initiated during the year prior to the search. We excluded any topics focusing on established medical treatments. Topics discussing possible triggers were included since some of the substances used for self-treatment also appeared to act as triggers for attacks potentially. In total, included topics were 32 (19 + 13). The forum reports were all written in the English language, and no translation of data was conducted.

**Table 1:** Searches with keywords “treatment cluster” for each discussion forum and limitation of searches

Discussion forum	Topics	Topics initiated last year	Included topics
Shroomery.org	409	23	7
Bluelight.org	152	27	4
Clusterbuster.org	161	12	8
Total included			19

**Table 2:** Searches with keywords “treatment migraines” for each discussion forum and the number of topics included in the analysis after limitation

Discussion forum	Topics	Topics initiated last year	Included topics
Shroomery.org	317	13	4 (3 duplicates)
Bluelight.org	242	42	8 (3 duplicates)
Clusterbuster.org	87	4	1
Total included			13

### Analysis

The collected reports were copied to a Word document (resulting in 56 pages of data), and the coding was performed manually. The analysis was carried out according to principles of thematic analysis and followed the different phases described by Braun and Clark [22]. In the first phase, the data was thoroughly read and re-read several times before initial coding. Next, the data were sorted into basic units of meaning. This step produced 411 coded elements (CE). Excerpts were created each time the underlying connotation of the text changed. The 411 CEs were further categorized into 63 categories by reviewing and analyzing characteristics and resemblances and by grouping related meanings together. Next, the level of abstraction was raised to differentiate present interrelationships within the 63 categories, generating six principal themes. Throughout the analysis, each theme was methodically revised and refined by continually returning to the original dataset for verification and support of the abstracted themes. The final themes and subsequent coding were then reviewed for consistency and audited individually by all authors. The themes and coding were also approved by two additional researchers experienced in thematic analysis.

## Results

The resulting six themes are presented below together with some illustrative quotations.

### A desperate need for effective treatments

This theme provides an insight into the difficulties that migraine and cluster headache sufferers experience and the typical incentives to use alternative therapies.

The pain caused primarily by CH but also some migraines was depicted as so immensely painful and disabling that sufferers were willing to “do anything” to alleviate them: “I have fractured multiple bones, and cluster pain is an order of magnitude worse.” Suicidal thoughts and feeling were reported as a result of the intense suffering and desperation caused by CH and severe cases of migraines. CH was sometimes labeled “suicide headache,” and this was something very relatable for many of the sufferers: “I came pretty close to ending my life over it.”

It was not only during the acute attacks that these conditions were causing difficulties. The sheer worry of the next debilitating attack was linked to anxiety and stress disorders: “Lots of cluster-headache sufferers end up with PTSD.” Also, sufferers of CH and severe migraines expressed how the disorder, as well as secondary diseases, complicated the routines of everyday life; everything from social contacts, work, and the ability to enjoy various activities was sometimes radically limited. Family and other relationships were potentially also heavily influenced: “Cluster headaches have broken up families, relationships, and marriages.”

CH patients often perceived themselves to be misdiagnosed by health care and felt their condition were not adequately treated. The conventional medical treatments were often described as virtually ineffective for CH: “I have tried everything with no success, including ergot derivatives, opiates, anticonvulsants, NSAIDs and so on.” Various opiate-based painkillers were commonly prescribed for CH sufferers but were predominantly reported as inadequate or even acting as potential triggers for attacks: “Opiates did nothing.”/“Opiates may well even trigger attacks as I am sensitive to histamine as a trigger.” Problems with addiction from the use of prescribed opiates further discouraged the use of opiate therapy: “I am 100 days sober off opiates for the first time in 5 years.”

There were some reports where medical personnel (i.e., physicians, psychiatrists) had advised an alternative or illegal treatment when current treatments were not sufficient: “My psychiatrist suggested that psilocybin-containing mushrooms might help.” A few reported prior use of illicit drugs, but those who never previously imagined taking an illegal drug, or do anything illegal, were also seeking alternative treatments from sheer desperation: “I cannot believe I have resorted to this, but nothing else works.”

### The role of the forum—finding alternative treatments and community support

This theme describes how CH and migraine sufferers used the discussion forums to find and exchange information regarding the use of alternative treatments and how to acquire the various substances employed for this purpose. Additionally, the forums were used as a platform to seek compassion, understanding, and fellowship: “One of the worst things that cluster sufferers go through is the feeling of being alone.” The support shared via these forums appeared to be highly valuable for this vulnerable population: “ When there is no hope to be found in professionals, online forums with people who have visited your private hell are sometimes all you have.” Similarly, the discussion forums were used by relatives and dependents of CH and migraine sufferers for support and information regarding these conditions.

Numerous users reportedly found effective treatments via the guidance from peers on these forums: “With the advice of one of these specific forums, I found the miracle drug.” The discussed substances often were fully illicit and could only be obtained from the black market or be self-produced. Other substances, so-called novel psychoactive substances (NPS), are semi-legal and were typically acquired from publicly available online vendors (gray market): “Various mail order companies are serving chemicals like these to the public.” Some of the online drug (NPS) vendors were recommended as somewhat knowledgeable of CH and substances used as potential treatment. A few fully legal substances were also considered.

Even when illegality was a factor, the availability of considered substances seemed relatively high. However, availability was also somewhat varying according to the legal situation in the respective country: “Being in Japan might add small challenges.” Some discussions revolved around how to bypass the limitations of the legal status to obtain various illegal drugs. For instance, using online “darknet” vendors was discussed as a way to acquire illicit substances, otherwise hard to find. Several sufferers stressed the importance of changes to drug laws or to make exceptions for some substances and conditions: “I really wish I lived in a state with accessible medical marijuana policies.” Another suggested route was to choose legal but equivalent or similar compounds.

Although the enthusiasm of those who experienced relief by various substances was apparent, the information exchange was often nuanced and focused on minimizing harm and to optimize the effectiveness of the self-treatments*:* “Synthetic tryptamines don’t have the records of safe medicinal and spiritual use that mushrooms have, promoting them to a novice seems like a poor idea.” Warnings concerning dangerous interactions with other drugs were issued. Especially a caution of combining prescribed antidepressants and serotonergic tryptamines was noted: “Be aware that the more serotonin agonists she is taking at increase the risk of developing serotonin syndrome.”

### Alternative treatment substances

A summary of substances and treatment alternatives used for self-treatment of CH or migraines is presented below. Recommendations on how to avoid certain substances, foods, and other factors possibly triggering attacks are also included in this theme.

Overall, the forum discussions revolved around general descriptions on the use of psychedelic tryptamines (not always specified which particular substance) to cure or alleviate these disorders: “Using psychedelics to treat migraines.”/“Treating cluster headaches with psychedelics.”

Psilocybin, or psilocybin-containing mushrooms, was commonly utilized for both migraines and CH: “I used magic mushrooms to abort my chronic migraines.”/“I am taking mushrooms for the treatment of cluster headaches.” The incentives and approaches to using psilocybin varied amongst sufferers; some initially used psilocybin for purposes outside the treatment of CH or migraines but were also pleased to experience alleviating effects on these conditions. However, most users did not appear to prefer any psychoactive effects and were solely seeking a possible alleviation of their ailment: “A toned down version of a mushroom trip may be very desirable in many contexts.”

LSD was a common and highly regarded substance for treating both CH and migraines in the reports: “LSD may be the most efficient of the psychedelic treatments.” The data also described other LSD-related substances; 1P-LSD or AL-LAD was mentioned as potential alternatives to LSD. Seeds from four different varieties of flowers, containing the tryptamine d-lysergic acid amide (LSA), like *Rivea corymbosa*, *Argyreia nervosa* (Hawaiian Baby Woodrose), or *Ipomoea tricolor* (Morning Glory) were also commonly used and recommended as a (mostly) legal and more available alternative.

Other psychedelic tryptamines were also frequently discussed as potential treatment options. Attempted self-medication using *N*,*N*-dimethyltryptamine (DMT), as well as various novel synthetic tryptamines, was described in several reports: “I have been dosing my girlfriend with 4-ACO-MET or 4-ACO-DMT. It aborted pain level 10 migraine attacks in 30 minutes or less that usually leaves her screaming incapacitated with pain.” Certain synthetic tryptamines were sometimes preferred over psilocybin (mushrooms) since the psychoactive effects were perceived as more manageable: “4-HO-MiPT and 4-HO-MET are said to be not as chaotic as shrooms”.

There were also some discussions on using various combinations of substances and how to test different combinations until the best possible effects were achieved: “The list includes a variety of ”exotic“ tryptamines but also many phenethylamines, particularly in the 2C- family.” A few also mentioned using combinations of prescription medications and non-approved drugs. The recommended administration of prescribed medications was sometimes altered by, for example, grounding pills to a powder to use by nasal insufflation or to exceed the prescribed dosage.

Cannabis was commonly discussed for its potential to alleviate symptoms or lessen the frequency of migraine attacks. Some had used cannabis for unrelated purposes but experienced additional benefits on the headaches.

Other substances, briefly mentioned as potential treatment alternatives, were melatonin, opium, ketamine, cocaine, lidocaine, and MDMA. Also, caffeinated energy drinks (or taurine that is present in most energy drinks) were mentioned: “Energy Drinks - Slam one right when you feel the attack coming on.” Vitamins and supplements were sometimes recommended but were not discussed extensively: “I am getting incredible results from being on the D3 regimen”. Other lifestyle factors like exercise, nutrients, and a healthy diet were also discussed and suggested: “Lots and lots of plant foods like broccoli or carrots and spinach.”

Discussions on preventing episodes of CH and migraines by avoiding certain “triggers” were present in the data. Alcohol, chocolate, fermented cheese, opiates, histamines, carbon oxide, carbon monoxide, sumatriptan, phenethylamines (2C− substances), sudden drops in blood pressure, and changes in weather were discussed amongst the suggested triggers to avoid: “Phenethylamines can trigger terrible migraines, especially 2C-series”/“Sumatriptan caused me to have 51 attacks in 7 days.”

### Dosage and regimens

Indicated dosages and discussions on dosing regimens are outlined in this theme. The timing and routes of administration were discussed for some substances. Principally, three different approaches or regimens for dosing were reviewed and recommended: the cyclic “busting” (or “clusterbuster”) method, frequent “microdosing,” or single and occasional “full” doses.

Generally, self-treatment was implemented according to one of the dosing regimens. Busting (or the “clusterbuster” method) is an administration regimen where psychedelic tryptamines are used in moderate to medium dosage and strategically timed with the regularly cyclic nature of CH episodes: “The use of psilocybin as a way to cure or manage cluster headaches, a.k.a. busting.” The dosage interval can differ between individuals; one example was dosing every fifth day during a cluster cycle until the cycle is over. Preventive doses are often used preceding a cycle to prohibit the onset of episodes or to reduce the intensity and or frequency of attacks. Discussions regarding the administration regimen “busting” did not clarify exact dosages, but generally, half the amount of a mild recreational dose was suggested. Busting regimen discussions focused more on the importance of the timing and interval of dosage: “Many have found the terror fades along with the prevention of complete cycles via busting.”

Microdosing was a related administration strategy frequently discussed and recommended. Microdosing is the practice of taking a sub-perceptual dose (an amount too small to produce typical “psychedelic” effects) of a substance: “The idea is to take enough to be effective against clusters without going on a significant trip.” The substances used for microdosing and were most commonly psilocybin, LSD, as well as LSA seeds, and some synthetic psychedelic tryptamines. Microdosing was utilized to avoid significant psychoactive effects, to enable more frequent use, and to prevent adverse effects: “Research thus far seems to indicate that microdosing is not harmful or dangerous.” Since apparent psychoactive effects did not hinder the daily routine, microdosing was sometimes preferred over the busting regimen*:* “I used the busting method for years but turned to microdosing, much easier to fit in.”

When not using a particular dosing regimen, it was typical to employ higher but single or occasional doses. For some individuals, higher or “full doses” were reportedly necessary to promote therapeutic effects. However, a “step-up” approach was typically recommended, starting with a tiny dose and gradually increasing the dosage until preferred effects were achieved.

A benchmark for occasional single doses of psilocybin was around 1 g of dry *Psilocybe cubensis* but could vary between 0.25 g and as much as 3 g. An ideal dose for one individual could be far too much for another. The preferred dosage varied with the sensitivity of the user and the desired effects:“ You might have to experiment with the dose a bit because what works for one person does not necessarily work for another.” The potency of the material and particular type of mushrooms also called for different dosage: “Around one gram of dried Cubensis is regularly used for a dose.” *P. cubensis* was the most common variety, but other species of mushrooms were also discussed: “With Psilocybe azurescens or Psilocybe cyanescens, 0,25 gram should be sufficient.”

The data contained a few discussions on various routes for administrating psilocybin, some suggested sublingual administration (ground up mushrooms under the tongue), and others preferred to mix the mushrooms with water or juice for drinking.

A preferred dosage of LSD had an interval between as less as 5 μg and over 150 μg, depending on personal preferences and if used occasionally or more frequently following a dosing regimen. It was common to use LSD quite infrequently; a few times a year was not an unusual practice: “Doses about once a year, started on 50ug and the same night re-dosed 50ug” and “ I think dosing 3-4 times a year will help me a lot”.

The dosage of LSA seeds was not extensively discussed, but it was suggested that around 50 seeds were needed for a full preventative dose, although it appeared more common to use less than 25 seeds and more frequently, following a dosing regimen. Mostly, the seeds were ingested whole, but occasional reports used various techniques to extract the active substances.

Exact dosages were mostly not defined regarding DMT, but usually a “full dose” was reportedly required for therapeutic effects on migraines or CH: “It would seem that a complete breakthrough hit is needed for a cure.” Also, for DMT, it was suggested that singular or infrequent dosage could have potential long-term beneficial effects on headache disorders: “Even a single dose, or perhaps a couple, can be a lifelong benefit.”

Other synthetic novel tryptamines like 4-AcO-DMT, 4HO-DMT, and 4-AcO-MET had suggested sub-psychedelic therapeutic dosages around 2–3 mg and 5-MeO-DALT around 12–15 mg.

Any particular dosage or administration methods for cannabis were not discussed; however, it was proposed that higher doses could have a triggering, rather than alleviating, effect: “Increase in migraine/headache intensity always goes hand in hand with an increase in dosage.” Also, the timing of cannabis use in relation to the attacks was discussed as a factor for successful treatment. Typically, it was recommended to use cannabis immediately when sensing the onset of an episode.

The few reports on lidocaine (Xylocaine) used doses around 25–30 mg in 5% solutions that were administrated through the nasal passage. One report suggested 500 mg taurine in a gel cap. Taurine was otherwise mostly used in energy drinks, and exact doses were not specified in the reports.

### Effects and treatment results

Effective treatment results, for both acute and prophylactic treatment, were reported for several of the substances concerned. (Adverse effects are discussed in the following theme.) Pre-eminently, the psychedelic tryptamines were described as remarkably effective and constituted a majority of the reports. For prophylactic treatment of CH, the psychedelic tryptamines were typically seen as the primary realistic option: “Only psychedelic treatments are shown to stop the recurrence of the cluster cycle.”

Overall, LSD and psilocybin were reported as highly effective for both CH and migraines. Both substances were reportedly effective for prophylactic as well as acute treatment. However, according to several reports, LSD possibly exhibits even higher potential for treating CH. The therapeutic potential of vaporized or smoked DMT seemed a bit more uncertain or complex compared to LSD or psilocybin: “DMT often helps, but sometimes makes it worse.” In one case, a full dose of DMT was effective and reportedly provided lasting prophylactic effects when all else (conventional medication, LSD, psilocybin, and so on) had failed*:* “For the first time in years, literally, I was not waking up with migraines anymore. Something happened in my brain that day.”

LSA seeds were said to have similar, but possibly less, effects than LSD and psilocybin: “HBWR seeds are not as useful as mushrooms.” The lack of results for some LSA users was sometimes accredited to the high variability in the potency of seeds, not always effective extraction techniques, and a tendency for under-dosing the seeds: “LSA was not actually working, I think I dosed too low, only used a few seeds at a time.” Seeds from *R. corymbosa* were described as the most efficient LSA containing seed, tough, successful treatment results were reported from other varieties as well: “I started to bust RC seeds and.....miracle. I can say that a total of 2 months of clusters in 5 years is an incredible success”.

Although not as prevalent as LSD or psilocybin, several other synthetic psychedelic tryptamines were discussed and reported as effective treatment alternatives: “I have had great success with acute treating of CH-attacks with 4-HO-MET, 4-AcO-DMT, 4-HO-MiPT, and 5-MeO-MiPT”. The LSD-analog AL-LAD had one report where it was effective for acute migraine treatment.

Microdosing was commonly reported as an effective treatment strategy, not only using psilocybin and LSD but also other psychedelic tryptamines like 4-ACO-DMT and 4HO-DMT. Microdosing appeared to be used for prophylactic effects primarily. Microdosing was reportedly a successful approach for most sufferers, but a few seemed to need fuller doses to have sufficient effects: “My partner could get away with taking sub-hallucinogenic doses to treat her cluster headaches, whereas I need a hallucinogenic dose to abort a migraine, which is unfortunate.”

The “busting” dosing regimen appeared to be an effective strategy for many sufferers: “Thank gosh busting preventatives are working.” Those using the “busting method” reported both acute and preventive treatment results, although it was described as crucial to follow a cyclic dosage scheme to obtain long-term results. Relapses were reported when the dosing regimen was not followed consistently: “Mostly pain-free, except for when I did not take my proper preventative dose.” The busting method was reportedly effective with LSD, psilocybin mushrooms, and various kinds of LSA containing seeds.

There were occasional reports where sufferers did not find relief or any beneficial effects from psychedelics at all. However, in these few cases, there was typically an uncertainty about dose or the potency of the material, and they were often based on single or a few treatment sessions: “I attempted to stop a cluster with what I thought would be an active (and my only) dose of mushrooms.”

The effects of self-treatment with cannabis appeared more contradictory and complex than other substances discussed. While some described expedient relief from the use of cannabis, others reported no benefits and some even found that cannabis could potentially trigger or intensify attacks (see the “Adverse effects” section). “I found out marijuana is awesome for migraines”/“It has done nothing.” Prophylactic long-term effects of regular cannabis use on migraines (not CH) with a lessening in the frequency of attacks were reported: “The weed actually does 100% keeps the tension migraines away for 2-3 months”. Facilitating sleeping during attacks and managing pain were other reported uses for cannabis. Also, it was described how the effects of cannabis served as a distractor from pain and other unpleasant sensations: “Weed helps me to sleep”/“Even when it does not cure the pain, it significantly lessens my care factor about it.”

One report described how cocaine could sometimes be used to stop ongoing CH attacks but did nothing to cure or reduce the frequency of episodes. Caffeinated energy drinks with taurine could also alleviate immediate symptoms: “Regarding Redbull, yes it works.” Melatonin was also occasionally discussed, but no alleviation of pain, or improvement of the conditions, was reported: “Melatonin did nothing for me.”

### Adverse effects

No severe adverse effects were reported, but there were some accounts of discomfort and temporarily increased symptoms and also some possible cases of remaining anxiety.

When using psilocybin, LSD, or DMT as an acute treatment, it was sometimes said to intensify pain and other symptoms initially, before any mitigating or preventative effects on CH or migraines were noticed: “I thought that the mushrooms hadn’t helped and I was back to where I started. But I haven’t had a headache since that night.” Psilocybin use was occasionally reported to cause anxiety or panic attacks. On the other hand, these adverse effects were also described as manageable by a more infrequent dosage interval by some of the same users: “I found that if I didn’t take shrooms more than once a month, I didn’t get anxiety.”

There were a few discussions on how treating migraines with LSD could increase the risk of developing sensory disturbances (hallucinogen-persisting perception disorder (HPPD)), especially for the sufferers of migraines with aura: “Seems people who have migraines with aura have a higher degree of HPPD after taking LSD.” However, no actual personal reports describing HPPD were present in the data.

A few reported increased perspirations and problems with focus and unexpected emotional experiences from microdosing with LSD or psilocybin, a: “I got the sweating too.” However, those experiencing this kind of perspiration were not sure if the sweating was accurately seen as an adverse effect. Since microdosing was often seen to produce beneficial effects like elevated mood, increased productivity, and an overall feeling of improved health, it was speculated that increased perspiration might be a part of some beneficial bodily process: “I am unsure whether the sweating was part of healing or just a quirky side effect.” The reports on the use of LSA containing seeds mentioned slight nausea but no other side effects*.*

For a few, cannabis appeared to trigger attacks potentially: “I get migraines/headaches almost every time I smoke.” Discussions on timing, dosage (see the “Dosage and regimens” section), frequency, and method of administration and especially the strain (the type of cannabis) or the quality of the product were actualized concerning eventual adverse effects or lack of benefits from cannabis use. “Ditchweed gives me migraines”//“The buds were not cured properly....they are too green.”

## Discussion

Our qualitative inquiry complements previous studies and illustrates the complex situation of treatment-resistant patients with headache disorders and how self-treatment is conducted. The result provides an insight into why, how, and by which substances and methods sufferers seek relief from CH and migraines. Furthermore, the result gives an appraisal of the potential effectiveness of commonly used substances and treatment strategies, as well as possible adverse effects. The central incentives to seek alternative treatments were described as deep feelings of dejection and despair from trying all available treatment methods from healthcare to little or no success. Also, the result shows how discussion forums are used to find community, support, and understanding in desperate and vulnerable circumstances. A reciprocal accumulation and evaluation of knowledge in this domain through the formation of Internet support groups and the promotion of harm reduction perspectives is also further highlighted through the present study.

Self-treatment with psychedelic tryptamines, primarily LSD and psilocybin, was reported to provide a significant lessening of the frequency and intensity of attacks in many cases of both CH and migraines. A full remission was also prevalently reported for both disorders. However, sufferers typically continued to use a psychedelic substance a few times a year to maintain their condition at a minimum. The findings largely confirm previous research [3, 6, 7, 9–12] indicating that psychedelic tryptamines appear effective for treatment of both CH and migraines, also in otherwise treatment-resistant patients.

The few individuals reporting no therapeutic effects from psychedelic tryptamines at all had typically only used these substances once or very few times. Therefore, several possible reasons for the lack of beneficial results were discussed on the forum, for example the timing or route of ingestion, dosage, and the potency of the material.

Self-treatment with cannabis was also commonly discussed, but treatment results were highly varying. While some reported acute relief or prophylactic benefits of cannabis use, others experienced a worsening of symptoms or even triggering of episodes. The differing results from using cannabis were discussed on the forums in relation to timing, frequency, and method of administration, dosage, and in particular the strain (type) of cannabis or the quality of the product. Since herbal cannabis consists of many different cannabinoids and other compounds, there might be active substances present potentially helpful for treating these conditions and other compounds exhibiting opposing effects.

Many other types of psychoactive substances, as well as supplements, vitamins, and herbal remedies, were discussed as potential remedies. However, all these were scarcely considered or only used in combination with other measures. Therefore, the effectiveness of these substances and supplements cannot be further addressed in the present study.

Treatment attempts were typically systematic rather than random, often following a particular dosing regimen. Principally, three different approaches or regimens for dosing psychedelic tryptamines were reviewed and recommended: (1) the cyclic “busting” (or “clusterbuster”) method, (2) frequent “microdosing,” or (3) single and occasional “full” doses. Microdosing was sometimes preferred (over “busting” or regular “full” doses) as it did not interfere too much with daily responsibilities and some also described additional beneficial effects like increased optimism, creativity, and awareness of self: “Microdosing alleviated my depression.” Some individuals reported insufficient therapeutic effects from using smaller more frequent doses but described how higher doses, with full psychedelic effects, had significant prophylactic effects for both CH and migraines. However, this population typically did not appear to have any interest in psychoactive effects, which were rather avoided by using sub-psychoactive doses or tolerated by those who acquired higher doses to achieve treatment results. Also, sufferers appeared to rather reluctantly use illegal substances out of sheer desperation and discussed how changes in drug laws or access to certain substances for certain conditions would be highly preferable.

Despite apparent dissatisfaction with established medicine and public policy, the forum discourse entailed scientific references and information from experts and medical practitioners as an addition to sharing personal experiences and reflections. Localized harm reduction perspectives, relevant to the specific type of drug board, have been identified as a key theme in drug-related forum discussions [20, 21, 23, 24], and this character of content was further observed in the present study. The participant’s personal needs for useful and objectively accurate information appeared to contribute to a collective process that produces relatively high-quality information focused on minimizing harm and to optimize the potential effectiveness of treatment attempts.

A prominent feature of the discussions was the heartfelt reports on the immense suffering and helplessness of CH sufferers who experienced frequent and debilitating pain and found little or no relief using available methods from healthcare. Several reports of misdiagnosis and how this motivated the sufferers to look elsewhere for information and possible relief were present in the data. The following quotation is a good representation of the point of view expressed by the many sufferers and the rationale of using these substances as a last resort: “Cluster headaches are so severe that doctor’s implicit prognosis is suicide or opiate addiction. One dose of LSD can treat this illness for up to a month. Ultimately, cluster headache sufferers who treat their condition with LSD often experience full remission and don’t have to use LSD again. So here we have a remedy that can treat this condition better than any other treatment and can potentially CURE cluster headaches! Yet, we let these patients commit suicide or get dependent on opiates for the rest of their lives*”.*

The intense and desperate situation expressed by many of the CH sufferers should be noted and taken most seriously as the desolation could sometimes lead to suicide or other harmful measures. It was observed in the present study how this desperation sometimes spurred risky behavior when obtaining and testing various treatment alternatives and how unregulated Internet vendors were used to obtain unknown and possibly harmful substances (NPS). NPS tryptamines like alpha-methyltryptamine (AMT) have caused poisonings with fatal outcome [25]. Several reports in the present study indicated that new and unknown substances (NPS) were used when LSD was hard to obtain. LSD and psilocybin are, when in pharmacological quality, not toxic, and deaths from the direct effects of LSD are unknown. However, when obtaining illicit substances like LSD, the risk of acquiring a mislabeled, adulterated, or impure substance is naturally present. In the present study, no severe adverse effects were noticed from attempted self-medication with these substances, but the long-term effects of such use are not known.

The role of hallucinogenesis (i.e., psychoactive/psychedelic effects) for the therapeutic potential of these substances has previously been addressed by researchers [3, 9] but is not yet fully explained. For example, the non-hallucinogenic ergot derivative, methysergide, was reported to be mostly ineffective for treatment of CH in the present study, and previous studies have indicated similar results [9]. On the other hand, the non-psychoactive ergot derivative BOL-148 was found to be equally effective as the psychoactive counterparts in some studies [9, 15]. Also, the psychedelic tryptamines were often reportedly effective at sub-psychoactive doses, both in the present study and previous studies. The aforementioned would suggest that hallucinogenesis is not needed for therapeutic effects on CH. No self-therapeutic use of BOL-148 was reported in the present study, most likely because of the unavailability of this substance.

### Limitations

The sample being self-selected and non-randomized may contribute to some bias, and the accuracy of individual reports cannot be guaranteed. However, the described effectiveness of psychedelic tryptamines does not appear to be based on selective reporting or drug romanticism. If skewed reporting was the culprit for the results, we suggest that the same would be presented for the uses of cannabis where the treatment results were reported as highly varying. Information on dosage was sometimes ill-defined or missing. Also, the purity or concentration of ingested materials is unknown. Therefore, the connection between dosage and effects could not be further elucidated in the present study.

The beneficial treatment results frequently appeared for both CH and migraines, but the nature of the data and methodology of the present study do not allow us to make any precise differentiations on the treatment response between the two disorders. Also, in several cases, sufferers reported being afflicted by several types of headaches, which further complicates any such conclusions: “I suffer from 3 different ones; tension migraines, TMJ migraines, and the wonderful cluster headaches.”

## Conclusions

Self-treatment of headache disorders is discussed in support groups online. Largely, this interest focuses on the use of the currently illegal psychoactive tryptamines, mainly psilocybin, LSD, and related substances. Often, this pursuit is driven by desperation, and these substances are considered a last resort. It was reported how several of the substances used can serve as potential treatments for migraine and CH. However, this population exposes themselves to risk by self-experimenting with illegal or sometimes new and unknown psychoactive substances. Given the vulnerability of this population, their situation is important to note and to consider seriously. This study also highlights the importance of the reciprocal knowledge production process and harm reduction content emerging from interactive drug forum discussions. More scientific studies are needed to develop safe and effective drugs. To minimize harm and to cater to the needs of this patient group changes or exceptions in legislation and other ethical considerations can be a required measure.

## Abbreviations

CAM: Complementary and alternative medicine
CE: Coded elements
CH: Cluster headache
DMT: *N*,*N*-dimethyltryptamine
HBWR: Hawaiian Baby Woodrose (LSA seeds)
HPPD: Hallucinogen-persisting perception disorder
ISG: Internet support groups
LSA: d-lysergic acid amide
LSD: Lysergic acid diethylamide
MDMA: 3,4-Methylenedioxymethamphetamine
NPS: Novel psychoactive substances
PTSD: Post-traumatic stress disorder
RC: *Rivea corymbosa* (LSA seeds)

## References

[1] Schuster NM, Vollbracht S, Rapoport A (2015). Emerging treatments for the primary headache disorders. Neurol Sci.

[2] Robbins MS, Starling AJ, Pringsheim TM, Becker WJ, Schwedt TJ (2016). Treatment of cluster headache: the American headache society evidence-based guidelines. Headache.

[3] Lambru G, Matharu M (2011). Serotonergic agents in the management of cluster headache. Curr Pain Headache Rep.

[4] Gooriah R, Buture A, Ahmed F (2015). Evidence-based treatments for cluster headache. Ther Clin Risk Manag.

[5] Schuh-Hofer S, Israel H, Neeb L, Reuter U, Arnold G (2007). The use of gabapentin in chronic cluster headache patients refractory to first-line therapy. Eur J Neurol.

[6] Tepper SJ, Stillman MJ (2013). Cluster headache: potential options for medically refractory patients (when all else fails). Headache.

[7] Sun-Edelstein C, Mauskop A. Alternative headache treatments: nutraceuticals, behavioral and physical treatments Headache Curr. 2011;51:697–705. 10.1111/j.1526-4610.2011.01846.x 21352222

[8] Barnes PM, Bloom B (2008). Complementary and alternative medicine use among adults and children: United States, 2007. Natl Health Stat Rep.

[9] Schindler EA, Gottschalk CH, Weil MJ, Shapiro RE, Wright DA, Sewell RA (2015). Indoleamine hallucinogens in cluster headache: results of the Clusterbusters medication use survey. J Psychoactive Drugs Routledge.

[10] McGeeney BE (2013). Cannabinoids and hallucinogens for headache. Headache.

[11] Govare A, Leroux E (2014). Licit and illicit drug use in cluster headache. Curr Pain Headache Rep.

[12] Di Lorenzo C, Coppola G, Di Lorenzo G, Bracaglia M, Rossi P, Pierelli F (2016). The use of illicit drugs as self-medication in the treatment of cluster headache: results from an Italian online survey. Cephalalgia.

[13] Johnson MW, Andrew Sewell R, Griffiths RR (2012). Psilocybin dose-dependently causes delayed, transient headaches in healthy volunteers. Drug Alcohol Depend.

[14] Majić T, Schmidt TT, Gallinat J (2015). Peak experiences and the afterglow phenomenon: when and how do therapeutic effects of hallucinogens depend on psychedelic experiences?. J Psychopharmacol.

[15] Halpern JH, Passie T, Huertas PE, Karst M (2009). Attack cessation and remission induction with 2-bromo-LSD for cluster headache. Cephalalgia.

[16] Mosek A, Dano M, Fainmesser Y, Hybloom Z (2014). EHMTI-0136. Medical cannabis in the treatment of treatment-resistant, chronic cluster headache. A retrospective Rep.

[17] Kjellgren A, Henningsson H, Soussan C (2013). Fascination and social togetherness—discussion about spice smoking on a Swedish Internet forum. Subst Abus.

[18] Soussan C, Kjellgren A (2014). The flip side of “spice”: the adverse effects of synthetic cannabinoids as discussed on a Swedish Internet forum. NAD Nord. Stud. Alcohol Drugs..

[19] Soussan C, Kjellgren A (2016). The users of novel psychoactive substances: online survey about their characteristics, attitudes and motivations. Int J Drug Policy.

[20] Andersson M, Kjellgren A. The slippery slope of flubromazolam: experiences of a novel psychoactive benzodiazepine as discussed on a Swedish online forum. NAD Nord Stud Alcohol Drugs. 2017:1–13. 10.1177/1455072517706304 PMC7450873 32934486

[21] Duxbury SW. Information creation on online drug forums: how drug use becomes moral on the margins of science. Curr Sociol 2015:1-18.

[22] Braun V, Clarke V, Braun V, Clarke V (2006). Using thematic analysis in psychology. Qual Res Psychol.

[23] Soussan C, Kjellgren A, Mazurkiewicz M, Głogowski M, Mrowińska D, Pakulski M (2014). Harm reduction and knowledge exchange—a qualitative analysis of drug-related Internet discussion forums. Harm Reduct J.

[24] Davey Z, Schifano F, Corazza O, Deluca P (2012). e-Psychonauts: conducting research in online drug forum communities. J Ment Health.

[25] Boland DM, Andollo W, Hime GW, Hearn WL (2005). Fatality due to acute alpha-methyltryptamine intoxication. J Anal Toxicol.

[26] Secretary’s Advisory Committee on Human Research Protections (2013). Considerations and recommendations concerning Internet research and human subjects research regulations, with revisions.

